# Absence of *Neisseria* *meningitidis* W-135 Electrophoretic Type 37 during the Hajj, 2002

**DOI:** 10.3201/eid0906.020725

**Published:** 2003-06

**Authors:** Annelies Wilder-Smith, Timothy M.S. Barkham, Suok Kai Chew, Nicholas I. Paton

**Affiliations:** * Tan Tock Seng Hospital, Singapore; † Ministry of Health, Singapore

**Keywords:** *N. meningitidis* W-15, Hajj pilgrimage, meningococcal disease, *N. meningitidis*, ET 37, dispatch

## Abstract

We document the absence of carriage of *Neisseria* *meningitidis* W-135 of the sequence type 11 in returning pilgrims after the Hajj 2002. This finding contrasts with the 15% carriage rate we previously reported in pilgrims returning from the Hajj 2001. The epidemiology of carriage may be changing or may have been controlled by vaccination and a policy of administering antibiotics to pilgrims from countries with a high incidence of meningococcal disease.

Two million pilgrims from all over the world congregate for the annual Islamic pilgrimage to Mecca and Medina in Saudi Arabia (Hajj pilgrimage). Because overcrowding facilitates person-to-person transmission of meningococci, this pilgrimage has been associated with meningococcal disease outbreaks (*1*–*3*). An international outbreak of serogroup W-135 meningococcal disease occurred during the Hajj pilgrimages in 2000 and 2001 (*4*,*5*). The outbreak-associated W-135 strains were of a single clone of the electrophoretic type (ET)-37 complex (*6*) and were closely related to other meningococci with an established propensity to cause disease clusters (*7*). Several reports from all over the world have shown that this W-135 outbreak strain affected not only pilgrims but also household contacts of returning pilgrims and the community at large, with the potential of non–Hajj-related further epidemics (*8*–*12*). As nasopharyngeal carriage is the primary source of transmission (*13*), worldwide dissemination is thought to be due to pilgrims who acquired W-135 carriage during the pilgrimage and introduced the strain on return to their countries of origin (*14*). We previously reported a 15% carriage rate of *Neisseria* *meningitidis* W-135 in Singaporean pilgrims returning from the Hajj 2001, despite the fact that all were vaccinated with quadrivalent polysaccharide meningococcal vaccine (*15*). W-135 carriage was substantially transmitted to unvaccinated household contacts (*15*,*16*). Pulsed-field gel electrophoresis (PFGE) of these isolates showed that they were distinguishable in 83% of the samples (unpub. data), indicating that this outbreak had a clonal origin. The high transmission rate of W-135 carriage from pilgrims to household contacts translated into a high attack rate of W-135 meningococcal disease among contacts in 2001 (*17*). We investigated carriage rates in pilgrims returning from the 2002 Hajj to determine whether W-135 was still a problem, and we compared the strains from 2002 with those from 2001 to document the evolving molecular epidemiology of Hajj-associated W-135 strains.

## The Study

We conducted a prospective study on the acquisition of meningococcal carriage in Singaporean Hajj pilgrims on the Hajj 2002. We used the same design and methods as we used in our previous study for the Hajj 2001, and the study was performed by the same research and laboratory staff (*15*). Pilgrims were recruited consecutively at the time of vaccination with quadrivalent meningococcal and influenza vaccine at a Muslim center that performs mass vaccinations for pilgrims referred by numerous national Muslim travel agencies. A swab sample was taken from both tonsils and the pharyngeal wall, by using a standard technique, and immediately transferred to a plate of selective culture medium (Oxoid GC, Basingstoke, UK). Repeat swab samples were taken 2 weeks after return from the pilgrimage. Returning pilgrims were asked if the symptoms of upper respiratory tract infection had occurred and if antibiotic drugs were taken during the pilgrimage, specifically a single dose of ciprofloxacin. All study participants gave written informed consent. The study was approved by the Ethics Committee of Tan Tock Seng Hospital.

Culture plates were immediately put in candle jars, transferred to the laboratory within 2 to 4 hours of collection, incubated at 37°C in humidified air with 5% carbon dioxide, and examined for bacterial growth at 24 and 48 hours (*18*). Identification of isolates as *N. meningitidis* was performed by traditional methods and was confirmed by API NH (BioMerieux SA, Lyon, France). The serogroup was determined by slide agglutination with polyvalent sera and serogroup-specific sera (A, B, C, D, Y, W-135, X, and Z) (Murex, Dartford, UK).

PFGE was performed on all meningococcal isolates by using previously described methods (*19*). The restriction enzyme used was *Spe*I. Multilocus sequence typing (MLST) was performed on all W-135 isolates, as well as on stored isolates from the 2001 Hajj, as described by Maiden et al. (*20*) by sequencing of seven housekeeping genes. Primers, determination of sequence alleles, and designation of sequence types are described on the MLST Web site (available from: URL: http://neisseria.org/nm/typing/mlst). Participant demographics, duration of stay at the Hajj, respiratory symptoms, antibiotic intake, carriage rates, and results of PFGE and MLST were compared with the findings by our group from Singaporean pilgrims returning from the Hajj 2001 (*15*).

## Conclusions

Tonsillopharyngeal swab samples were taken from 193 Malay pilgrims at a median time of 30 days (range 18–52) before their departure for the Hajj. One hundred fifty-three (79%) had a repeat swab sample taken at a median time of 10 days (range 2–17) after their return from the Hajj. The mean age was 48 (SD 8.07) years, and 48% were male. The mean duration of stay was 33 days (range 14–41, SD 3.6). Returning pilgrims reported cough (70%) and use of antibiotics (52.9%). Nine percent of pilgrims reported ciprofloxacin use.

Four of the pilgrims (2.6%) were carriers before the Hajj. Three isolates could not be grouped; one was serogroup B. Two (1.3%) of the returning pilgrims were carriers, and both isolates were serogroup W-135. The PFGE patterns of the two isolates of W-135 differed from each other by >10 bands, and each differed by >7 bands from the predominant PFGE pattern identified in the 2001 Hajj (*15*).

On MLST, these two isolates were sequence type (ST)-192 and ST-32 and shared only one of seven alleles with each other. They shared none of the seven alleles with the isolates from the 2001 Hajj. Thirty (94%) of the 32 isolates from the 2001 Hajj pilgrims were ST-11. The table summarizes the results from the 2002 versus 2001 Hajj pilgrimage.

**Table:** Characteristics of pilgrims returning from the Hajj 2002 compared with those returning from the Hajj 2001^a^

Characteristics	Returning Hajj pilgrims 2002 (n=153)	Returning Hajj pilgrims 2001 (n=171) (ref. 15)	p value
Median age (yrs)	48	48	
Gender (male) (%)	48	46	
Race (Malay) (%)	98	98	
Median interval between return from pilgrimage and throat swab in days (range)	10 (2–17)	17 (1–45)	
Median duration of pilgrimage in days (range)	33 (14–41)	33 (3–47)	
Overall meningococcal carriage rate (%)	1.3	17	<0.001
Carriage of W-135 ST 11 (%)	0	15	<0.001
Cough (%)	75	56	NS
Antibiotic drug use (%)	56	41	NS

^a^NS, not significant.

We documented a low W-135 meningococcal carriage rate (1.3%) in pilgrims returning from the 2002 Hajj, which is in stark contrast to the 15% carriage rate in pilgrims returning from the year 2001 Hajj (*15*). On multilocus sequence typing, these isolates were ST-192 and ST-32 and not ST-11, which was the dominant sequence type in returning pilgrims from the year 2001 Hajj. ST-11 is most commonly associated with the hypervirulent ET-37 complex (*6*,*7*). Our strains from the year 2001 Hajj are therefore highly likely to be the same as the ET 37 ST-11 responsible for the outbreak in Saudi Arabia (*6*,*7*,*9*). The two W-135 strains (ST-192 and ST-32) isolated in pilgrims returning from the 2002 Hajj were clearly distinct, both in PFGE and MLST, from the W-135 strain (ST-11) isolated in pilgrims returning from the year 2001 Hajj (*15*) and are thus unlikely to have evolved from ST-11 strains circulating in 2001. Genetically distinct Hajj-compatible phenotypes were also reported in France in 2002 (*21*).

While carriage rates are indicative of the potential of a meningococcal outbreak, occurrence of disease is ultimately more informative (*21*). The absence of the hypervirulent W-135 ET ST-11 in returning Singaporean pilgrims from the Hajj 2002 is reflected by an absence of Hajj-associated clinical cases of W-135 disease in Singapore in 2002. This finding is also consistent with international reports that showed a marked decrease in cases of meningococcal disease in 2002 caused by the Hajj 2000/2001 outbreak strain (*21*). Therefore, in 2002, the W-135 carriage was probably also low in pilgrim populations other than these from Singapore.

The marked difference in W-135 carriage rates is unlikely to be due to selection bias or study methods, as we used the same recruitment strategy, swabbing techniques, and laboratory methods in 2002 and 2001. Although the decrease in carriage in the year 2002 could reflect spontaneous changes in epidemiologic features of the outbreak, a number of public health interventions may have played a role. Influenza vaccination was given in the 2002 cohort but not in the 2001 cohort, and respiratory symptoms are known to promote meningococcal transmission (*22*). However, these interventions are unlikely to account for the difference in carriage: frequency of upper respiratory symptoms were similar (or even more frequent) in the pilgrims of 2002 compared to the pilgrims of 2001. More rigorously enforcing a long-standing policy to administer antibiotics to pilgrims from Africa upon entry to Saudi Arabia (*5*), plus the recent extension of this policy to incoming pilgrims from the Indian subcontinent (*23*), may have played a role in decreasing the importation of W-135 to the pilgrim pool. In addition, more liberal use of ciprofloxacin may have occurred during the 2002 Hajj. Nine percent of the pilgrims in our cohort reported ciprofloxacin use.

However, the main difference between the year 2002 and 2001 Hajj pilgrimages was that coverage with quadrivalent meningococcal polysaccharide vaccine was only partial in 2001 (*9*), whereas almost complete vaccine coverage for this pilgrimage can be assumed for the year 2002 after this vaccine became a Hajj visa requirement for all pilgrims (*21*). Although polysaccharide vaccine does not prevent acquisition of carriage (*13*), as confirmed in our 2001 cohort (*15*), polysaccharide vaccines can induce transient reduction of carriage (*24*). In addition, vaccines reduce the incidence of meningococcal disease and thus circulation of meningococci. Almost complete vaccine coverage of 2 million pilgrims may have therefore contributed to a decrease in the spread of carriage within this pilgrim population and consequently reduced transmission of carriage from returning pilgrims to their contacts.

The significant decrease of W-135 meningococcal carriage and the absence of ST-11 in returning pilgrims from the Hajj 2002 are important findings with regard to public health policy. Our documented low W-135 carriage rate in Singaporean pilgrims in the year 2002 is indicative of a decreased potential for spread of meningococcal disease in close contacts of returning pilgrims. On the basis of the high carriage rate in returning pilgrims, together with the transmission to household contacts and observed secondary cases in the community in 2001 (*16*,*17*), we suggested that administrating antibiotics to returning pilgrims would be appropriate (*15*). Indeed, the Saudi Arabia authorities have implemented such a policy for their returning Saudi pilgrims (*23*). However, the epidemiologic features of carriage may be changing or has been controlled by vaccination and administering antibiotic drugs to incoming pilgrims from countries with high incidence. Thus, the administration of antibiotics to all returning pilgrims appears to be unnecessary at the present time. Ongoing surveillance of carriage rates both in the resident population in Saudi Arabia as well as in arriving and departing pilgrims is paramount for rapid readjustment of a policy to administer antibiotics to eradicate carriage.
